# Intramural Ganglion Structures in Esophageal Atresia: A Morphologic and Immunohistochemical Study

**DOI:** 10.1155/2009/695837

**Published:** 2009-07-27

**Authors:** Biagio Zuccarello, Antonella Spada, Nunzio Turiaco, Daniela Villari, Saveria Parisi, Isabella Francica, Carmine Fazzari, Federica Pederiva, Juan A. Tovar

**Affiliations:** ^1^Policlinico Universitario G.Martino, 98125 Messina, Italy; ^2^Policlinico Universitario, Padova, Italy; ^3^Hospital Universitario La Paz, Madrid, Spain

## Abstract

*Introduction and Aim*. Disorders of esophageal motility causing dysphagia and gastroesophageal reflux are frequent in survivors to esophageal atresia (EA) and distal tracheoesophageal fistula (TEF). The aim of the present study was to investigate the histologic and immunohistochemical features in both esophageal atretic segments to further understand the nature of the motor disorders observed in these patients. *Material and Methods*. Esophageal specimens from 12 newborns with EA/TEF and 5 newborns dead of unrelated causes were examined. The specimens were fixed in 5% buffered formalin, included in paraffin and cut in 5 micron sections that were stained with hematoxilin and eosin (H and E), and immunohistochemical stainings for Actin, S-100 protein, Neurofilament, Neuron-Specific-Enolase, Chromogranin A and Peripherin were evaluated under the microscope. *Results*. In controls, the distribution of the neural elements was rather homogenous at both levels of the esophagus. In contrast, the atretic segments showed quantitative and qualitative differences between them with sparser nervous tissue in the distal one in comparison with the proximal one and with controls. *Conclusions*. These results further support the assumption that histomorphological alterations of the muscular and nervous elements within the esophageal wall might contribute to esophageal dysmotility in patients surviving neonatal operations for EA/TEF.

## 1. Introduction

Esophageal motor disorders are common following successful repair of esophageal atresia and tracheoesophgeal fistula (EA/TEF) [1–3]. Dysphagia, gastroesophageal reflux, altered or absent peristaltis in the affected esophagus were documented by clinical, radiological, isotopic, endoscopic and pH-manometric observations [4–8]. In the past, this was attributed to a neurologic defect, probably due to partial denervation of the esophagus during operative dissection. Previously, we documented disorders of the esophageal motor activity in both untouched esophageal atretic segments on patients with EA/TEF or isolated EA by preoperative endoluminal esophageal manometry [9]. These motor anomalies were confirmed by histological and immunohistochemical studies in human [10–14] and animal models [15–21] suggesting that abnormal innervation and neuromuscular defect of the esophagus are prexisting to the surgery. The aim of the present study was to investigate the histologic and immunohistochemical characteristics of the nerve cells, fibers and bundles in both esophageal atretic segments to further enlightening the nature of the motor disorders observed in these patients. 

## 2. Material and Methods

Twelve newborns (7 boys and 5 girls, with the average gestational age 36.1 ± 2.21 days and postnatal age of 3.25 ± 1.95 days) with EA/TEF (type C without long gap) were examined. The specimens were obtained from the proximal esophageal segment in 9 cases and from the distal segment in 10 cases at the time (at birth) of the primary anastomosis. For comparison, 5 esophageal samples were obtained from autopsies of babies dead of unrelated causes, 1 to 2 mm above the bifurcation of the trachea. 

 Histological and immunohistochemical analyses were performed in all cases on formalin-fixed, paraffin-embedded tissues. Serial sections (4-5 micron) from the distal end of the upper esophageal pouch (UEP), the distal esophageal segment (DES) and it's the corresponding counterparts from controls were cut and stained with Hematoxylin and Eosin (H and E). We also used a immunohistochemical panel (IHC) with primary monoclonal and polyclonal antibodies. The sections were heated in citrate buffer (Antigen Retrieval Citrate buffer pH 6.0) for 30 minutes in a high-power microwave oven (400 W) to perform antigen retrieval. After rinsing in phosphate buffered saline solution (PBS, pH 7.4), endogenous peroxidase activity was blocked in 3% hydrogen peroxide for five minutes. Immunohistochemical procedures were carried out employing the following primary antibodies. 

(1) Anti-A (Actin) mouse monoclonal antibody clone asm 1 (1A4) Ylem Products S.r.l. Avezzano, Italy). Actin is a globular structural protein and represents the monomeric subunit of citosolic microfilaments and of thin filaments which are part of the contractile apparatus in muscle cells (actomyosin myofibrils). 

 (2) Anti S-100 Protein rabbit polyclonal antibody diluted 1 : 600 Novocastra Laboratories Ltd, Belliol Business Park West, Newcastle (UK). S-100 Protein is a protein that is present in high concentration in glial and Schwann cells in which it modulates a wide range of intracellular processes, including contraction and intracellular signal transduction and it is involved in promoting axonal growth, glial proliferation and neuronal differentiation. The Anti S-100 Protein represents an unequivocal neuroepithelial istogenetic marker to evaluate the neuroectodermal cell-lines, including ganglion cells/Schwann cells. 

 (3) Anti NFs (Neurofilament) mouse monoclonal antibody (clone NR4/RT97), diluted 1 : 100 Ylem Products S.r.l Avezzano, Italy. Neurofilament is a type IV intermediate filament founded specifically in axons of mature large neural cells. It probably provides a structural support for neurons and synapses. The NF immunostaining was used to evaluate the presence of myenteric plexus in normal and atretic oesophageal segments and to detect the neurogangliar structures. 

 (4) Anti-P (Peripherin) mouse monoclonal antibody (clone PJM 50), diluted 1 : 100 Novocastra Laboratories Ltd, Belliol Business Park West, Newcastle (UK). Peripherin is a type III intermediate filament protein expressed in mature and developing peripheral neurons, including enteric ganglion cells. By revealing biological processes of native cells and tissue allows to visualize, localize, and quantify endogenous protein interactions and modifications. 

 (5) Anti-NSE (Neuron Specific Enolase) mouse monoclonal antibody (clone BBS/NC/VI-H14), diluted 1 : 100 Santa Cruz Biotechnology, CA, USA. The cytosolic Enolase includes different, widely distributed glycolytic dimeric enzymes that catalyze the interconversion of 2-phosphoglycerate and phosphoenolpyruvate. The isoenzymes of enolase are mainly founded in neurons and neuroendocrine cells. 

 (6) Anti CgA (Chromogranin A) mouse monoclonal antibody (clone DAK-A3), diluted 1:50 Dako A/S, Glostrup Denmark. Chromogranin A is the major member of the granin acidic glycoproteins family that plays multiple roles in the process of regulated neurotransmitters. Cga immunostaining decores myenteric ganglion cells.

 The IHC techniques of S-100 polyclonal and NF monoclonal antibodies were used in both participating institutions.

The sections were incubated with primary antibodies at 4°C overnight in a moist chamber. After being rinsed in PBS, the slides were reincubated for 10 minutes at room temperature with a biotinylated link universal secondary antibody, and developed in 3.3′-diaminobenzidine-HCl. 

 After a weak haematoxylin nuclear counterstaining, the sections were finally dehydrated and mounted in a synthetic medium. 

 To evaluate the ganglion cells in each case, five visual fields that consisted of myenteric and submucous plexuses were examined under microscope.

## 3. Results (Table 1)

### 3.1. Histology

In the control group, the distribution of all elements was rather homogenous in both proximal and distal portions of the esophagus. In contrast, the atretic segments had hyperplastic lining epithelium, oedema and disarranged muscle bundles with fibrosis in the UEP. Additionaly, the DES had mucosal and muscular hypoplasia, abnormal submuscosal elastic fibers and disorganization of muscularis mucosae and muscularis propria.

### 3.2. Immunohistochemistry

**Figure 1:**
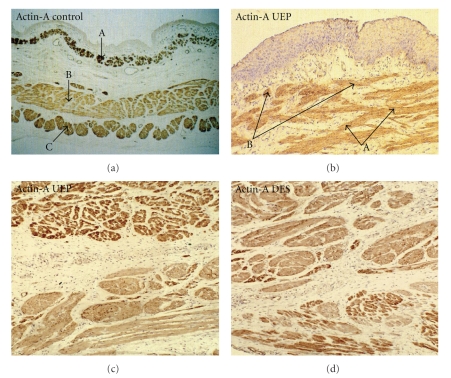
Actin-A. (a) Control neonatal esophagus: Anti-A immunostaining marks strongly the muscularis mucosae (A), as well as both the inner (B) and outer (C) muscle layers; (b) Presence of disarranged and fragmented muscle bundles (A) reaching the epithelial basal line and segmental agenesia of muscularis mucosae. (B) Anti-A immunostaining is reduced in the smooth musle bundles of the UEP (c), but it is weaker in (d) the DES.

**Figure 2:**
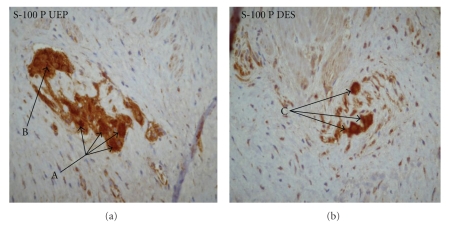
S-100 Protein (S-100). (a) The UEP shows fewer ganglion cells in the myenteric plexus (A) and an increased immunoreactivity for S-100 (B) than the control; (b) The DES shows few small immature cells (C) with less marked immunostaining than in the UEP.

**Figure 3:**
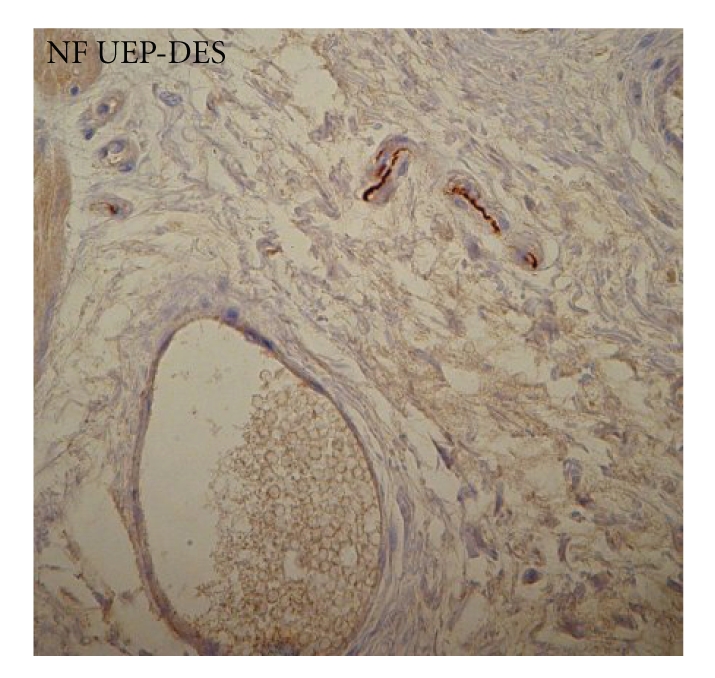
Neurofilament (NF). A diffuse weak immunostaining expression is obvious at myoenteric site of both atretic esophageal segments. In some cases the NF immunoreactivity is absent in the DES.

**Figure 4:**
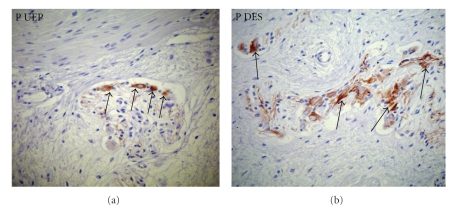
Peripherin (P). Immunoreactivity is less marked in the myoenteric ganglion cells of (a) the UEP than in (b) the DES.

**Figure 5:**
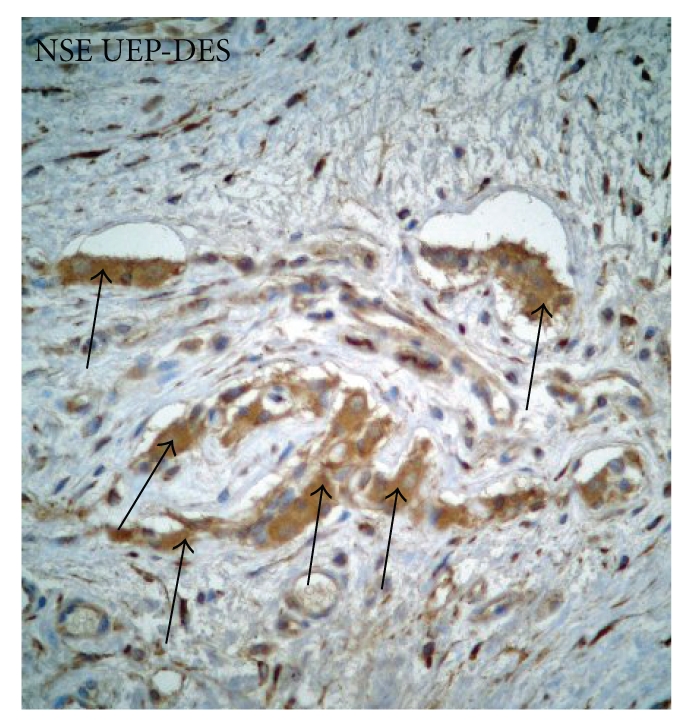
Neuron Specific Enolase (NSE). Weak cytoplasmic positivity in both proximal and distal segments.

**Figure 6:**
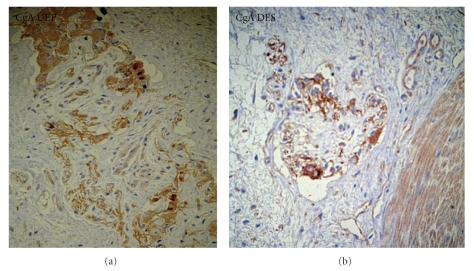
Chromogranin A(CgA). Positive immunoreactivity is more marked in (a) the UEP than in (b) the DES.

(1) Actin. The control exhibited a well defined muscularis mucosae which was longitudinally oriented and outer muscle bundles mainly consisted of skeletal dense fibers by mutual touching. In the UEP the pathological features displayed agenesia of muscularis mucosae that was extensively replaced by a soft fibrous network and an evident disarrangement of muscle bundles. Moreover, a complete disorganization of muscularis mucosae and muscularis propria was present in the DES. Various sized muscle bundles were seen, sometimes reaching the epithelial basal line. The transversal muscular layer was partially tickened and contained both smooth and striated contractile cells. In the DES the outer longitudinal muscle layer was partially loose and thinner than observed in the control (Figure 1). 

 (2) S-100 Protein. In this study an increased S-100 cells expression was evident and prevalent in the muscle layer of the UEP as computed to the control and distal segment (Figure 2). Moreover, the atretic segments showed a reduced number of ganglion cells and the presence, in the DES, of few small immature neural cells. This feature denotes that the intrinsic esophageal innervation is deficient. 

 (3) In our study NF-positive reaction was localized in the myenteric plexus of normal esophagus and was less intensely reactive in the UEP and weak or absent in the DES (Figure 3): this weak or negative NF immunostaining reveals a diffuse neural dismaturity inside the esophageal wall. 

 (4) In this study myoenteric ganglion cells strongly express P in the DES, as compared to esophageal controls; the immunoreactivity for P is less marked in the UEP. This finding denotes regenerative events of enteric ganglion cells. This finding is coherent with a parallel defective NF expression by the same neuroepithelial cell line (Figure 4). 

 (5) In our cases the NSE immunoreactivity showed poor or negative espression in both atretic segments in EA/TEF group (Figure 5). This negative immunoreactivity is significant for a deficit of the neoglycogenesis. 

 (6) In the present study we observed, in atretic segments, a positive immunoreactivity more marked in the UES than in the DES (Figure 6). A reduced expression of immunoreactive CgA in ganglion could be significant for a defective neurotransmitter release.

## 4. Discussion

Despite a significant progressive improvement in the management of EA/TEF has been achieved, cause of refinements of surgical and anesthesiologic techniques and neonatal intensive care [1–3], the symptoms related to esophageal dysmotility (dysphagia, gastroesophageal reflux disease, altered or absent peristaltis) are common following successful repair of EA/TEF [4]. In the past, several authors have pointed out the presence of motor disorders in the esophageal body and sphyncters on EA/TEF symptomatic or asymptomatic survivors: cinefluorographic and isotopic studies showed altered esophageal motility also in the long-term follow-up [5, 6]; pH-metry revealed the presence of gastroesophageal reflux [7]; manometry showed abnormal spastic esophageal zones, incomplete relaxation of the upper esophageal sphincter, bifasic peristaltic waves in the esophageal body and, also, hypotension of the lower esophageal sphincter [8, 9]. 

 Esophageal innervation has been investigated in EA/TEF to clarify the altered esophageal peristalsis. Nakazato et al. studying the Auerbach plexus in EA/TEF with H and E, showed a markedly lower relative amount of neural tissue, especially in the DES, larger ganglia and ticker interganglionic fibers in the UEP, a looser than normal Auerbach plexus in the DES and to a lesser degree in the UEP, suggesting the existence of congenital functional impairment due to abnormal development of the myenteric plexus [10]. 

 Immunohistochemical studies with Actin showed agenesia of muscularis mucosae and an evident disarrangement of muscle bundles in the UEP [11] and, histologically, complete disorganization of muscularis mucosae and muscularis propria in the DES [12]. From the above findings, it can be inferred that EA/TEF is accompanied by a development failure of some mesodermal-derived buds in connection with a diagonal deviation of the esophago-tracheal septum. The finding of disarranged transversal muscle layer for the immunoreactive to actinin antibodies support this point of view and it is also in agreement with ganglion and nerve thinning out. 

 Recently, other authors studied EA/TEF by histology and immunohistochemistry in human and in an animal model. Histopathologic studies with H and E were conducted on an Adriamycin treated animal model to determine how closely it resembles the human pattern. In the rat model immunohistopathological studies found there were significant abnormalities of the intramural nervous components of the oesophagus, involving both the excitatory and inhibitory intramural nerves and the distribution of nerve fibers in the plane of the myenteric plexus in the atretic esophagus is deficient [15–21]. These patterns suggested that these abnormalities may underlie the esophageal dismotility clinically showed by infant with EA. Boleken et al. showed, in the UEP of human EA/TEF, a deficiency of distribution of neural tissue and elevated expression of S100, as a fine fibrillary pattern, in the muscular layer and myenteric plexus which stains for glial cells; moreover, the NF immunoreactivity was significantly reduced [13]. As this finding was not detected with NF stain, the increased cells were thought to be Schwannian in origin, and the hypertrophied glial tissue might signify a compensation for defective neuronal tissue. 

 In the present study we confirmed the findings of Boleken et al. evidenced in the UEP, while we showed that the deficiency of distribution of neural tissue is more marked in the DES. We observed the similar defective expression for NF in the UEP but it, in some instances, is absent in the DES. The NF immunostaining is a specific stain for mature neurofilament, which means a defective or absent maturation of nerve cells. 

 For this purpose, we used the Peripherin (P) stain observing an immunoreactivity more marked in the DES than in the UEP; this finding denotes an early maturation level of enteric ganglion cells(neuroblasts). It is coherent with a parallel defective NF expression by the same neuroepithelial cell line. The specificity of the P indicates that this intermediate filament protein has potential as a marker for this related neural crest neuropathy. Li et al. showed, in the DES, decreased staining of NSE (and substance P) and increased staining of VIP (and NOS): the imbalanced excretion of neurotrasmitters in the intramuscular motor nerve endings and the abnormal intrinsic dysplasia of myenteric nerve plexus, were the main characteristics of abnormal intrinsic innervation, which may be responsible for the postoperative dysfunction of EA/TEF [14]. 

In the present study, we also confirmed the previous innervation patterns described by Li et al. for the NSE immnoreactivity and we observed a decreased or negative cytoplasmic positivity in both proximal and distal segments. This negative immunoreactivity is significant for a deficit of neoglycogenesis. 

 CgA has been used as an immunohistochemical marker for normal and pathological neuroendocrine tissues: the physiologic role of this protein has not been fully elucidated, but it has been hypothesized that it may serve as a precursor to other biologically active peptides or may serve as a function in the intracellular production of hormones and neuropeptides. We used the CgA stain observing a positive immunoreactivity more marked in the UEP than in the DES; the reduced Cga expression in the DES might be significant for incomplete activity of neurotransmitter release. 

In conclusion, our histological and immunohistochemical studies partially confirmed the previous observations, adding at the same time new possibility to study in depth the UEP and DES segments. In fact, the present investigation on the atretic segments of EA/TEF, specially the DES, showed, with the P-immunostaining, the presence of immature neural cells (neuroblasts) and, with the CgA immunoreactivity, a granin deficit with subsequent altered activity of neurotransmitter release. These findings indicate a delay in neuronal differentiation and myenteric plexus organization that helps to explain the esophageal motility disorders seen before surgery in patients with EA/TEF, and might play a role in the observed postnatal oesophageal dismotility. Despite this new finding, the etiology of the motor disorders remains still unclear. However, further researches are needed to assess more precisely the exact nature of the esophageal innervation and neuromotor abnormalities.

## Figures and Tables

**Table 1:** Summary of morphologic and immunohistochemical findings of the esophageal segments in human EA/TEF.

Tissue	Proximal segment (UEP)	Distal segment (DES)
Mucosa	Epithelial hyperplasia	Epithelial hypoplasia
	Dysplasia and dystrophy	Dysplasia and dytrophy
	Reduction of muscularis mucosae	Absence of muscularis mucosae

Submucosa	Hyperplasia of elastic fibers	Hypoplasia of elastic fibers
	Infiltration of myofibrilles	Dysplasia and dystrophy
	Dysplasia and dystrophy	

Circular musculature	Muscular hypoplasia	Muscular hypoplasia
	Fragmentation of the myofibrilles	Disorganization of the myofibrilles

Intermuscular collagen and elastic tissues	Connective fibrosis	Connective fibrosis
	Endomisial fibrillogenesis	Endomisial fibrillogenesis

Myoenteric plexus of auerbach	Reduction of neurocells	Reduction of neurocells
	Increase of Schwann's cells	Increase of Schwann's cells
	Immaturity of neurocells	Immaturity of neurocells

Longitudinal musculature	Muscular hypoplasia	Muscular hypoplasia
	Fragmentation of the myofibrilles	Disorganization of the myofibrilles
